# Automatic quantitative computed tomography measurement of longitudinal lung volume loss in interstitial lung diseases

**DOI:** 10.1007/s00330-021-08482-9

**Published:** 2022-01-14

**Authors:** Salim A. Si-Mohamed, Mouhamad Nasser, Marion Colevray, Olivier Nempont, Pierre-Jean Lartaud, Anna Vlachomitrou, Thomas Broussaud, Kais Ahmad, Julie Traclet, Vincent Cottin, Loic Boussel

**Affiliations:** 1 Radiology Department, Department of Cardiovascular and Thoracic Radiology, CHU Cardiologique Louis Pradel, Louis Pradel Hospital, 59 Boulevard Pinel, 69500 Bron, France; 2 University of Lyon, University Claude-Bernard Lyon 1, UJM-Saint-Étienne, CNRS, Inserm, CREATIS UMR 5220, U1206, 69621 Lyon, France; 3 National Reference Center for Rare Pulmonary Diseases, Louis Pradel Hospital, Hospices Civils de Lyon, UMR 754, INRAE, Claude Bernard University Lyon 1, Lyon, France; 4 Radiology Department, Hôpital de La Croix-Rousse, 103 Grande rue de la Croix Rousse, 69004 Lyon, France; 5 Philips Research, Suresnes, France

**Keywords:** Tomography, Lung disease, interstitial, Pulmonary fibrosis, Idiopathic pulmonary fibrosis, Deep learning

## Abstract

**Objectives:**

To compare the lung CT volume (CT*vol*) and pulmonary function tests in an interstitial lung disease (ILD) population. Then to evaluate the CT*vol* loss between idiopathic pulmonary fibrosis (IPF) and non-IPF and explore a prognostic value of annual CT*vol* loss in IPF.

**Methods:**

We conducted in an expert center a retrospective study between 2005 and 2018 on consecutive patients with ILD. CT*vol* was measured automatically using commercial software based on a deep learning algorithm. In the first group, Spearman correlation coefficients (*r*) between forced vital capacity (FVC), total lung capacity (TLC), and CT*vol* were calculated. In a second group, annual CT*vol* loss was calculated using linear regression analysis and compared with the Mann–Whitney test. In a last group of IPF patients, annual CT*vol* loss was calculated between baseline and 1-year CTs for investigating with the Youden index a prognostic value of major adverse event at 3 years. Univariate and log-rank tests were calculated.

**Results:**

In total, 560 patients (4610 CTs) were analyzed. For 1171 CTs, CT*vol* was correlated with FVC (*r*: 0.86) and TLC (*r*: 0.84) (*p* < 0.0001). In 408 patients (3332 CT), median annual CT*vol* loss was 155.7 mL in IPF versus 50.7 mL in non-IPF (*p* < 0.0001) over 5.03 years. In 73 IPF patients, a relative annual CT*vol* loss of 7.9% was associated with major adverse events (log-rank, *p* < 0.0001) in univariate analysis (*p* < 0.001).

**Conclusions:**

Automated lung CT volume may be an alternative or a complementary biomarker to pulmonary function tests for the assessment of lung volume loss in ILD.

**Key Points:**

• *There is a good correlation between lung CT volume and forced vital capacity, as well as for with total lung capacity measurements (r of 0.86 and 0.84 respectively, p* < *0.0001).*

• *Median annual CT volume loss is significantly higher in patients with idiopathic pulmonary fibrosis than in patients with other fibrotic interstitial lung diseases (155.7 versus 50.7 mL, p* < *0.0001).*

• *In idiopathic pulmonary fibrosis, a relative annual CT volume loss higher than 9.4% is associated with a significantly reduced mean survival time at 2.0 years versus 2.8 years (log-rank, p* < *0.0001).*

## Introduction

Interstitial lung diseases (ILDs) encompass a heterogeneous group of chronic and fibrotic lung diseases with distinct disease course and prognosis [1]. They may be associated with progressive lung volume loss with impaired quality of life, and in advanced stage, respiratory failure. Idiopathic pulmonary fibrosis (IPF), an inexorably progressive disease, is the most severe and lethal among others [2, 3]. Survival is shortened in patients with lower forced vital capacity (FVC) at baseline and annual FVC decline ≥ 10%. FVC has been thus proposed as a surrogate marker for disease progression and mortality in all ILDs and has been advocated as a primary outcome in major clinical trials in IPF [4–6]. However, FVC measurement is subject to inherent measurement variability and might be inaccurate in frail patients, advanced disease stages, and subjects with intractable cough [7, 8]*.* Therefore, a new feasible, reproducible, and effortless surrogate biomarker is still needed.

Chest high-resolution computed tomography (HRCT) is mandatory for disease evaluation in patients with ILD and is used for diagnostic, monitoring, and prognostic purposes. The current guidelines recommend that pulmonary function tests (PFT) and chest HRCT are both fundamental in patient follow-up [9–11]. Moreover, CT loss volume derived from visual or automated quantification of the lung volumes on HRCT has shown great interest for assessing the degree of severity, disease progression, and mortality in IPF and systemic sclerosis-associated ILD, in a relatively small ILD population [12–15]. Yet, there is a dearth of data on the role of longitudinal CT lung volume loss and annual decline and its prognostic in the IPF population.

Therefore, using a newly commercially available deep learning algorithm for automatic quantification of lung CT volume, we compare the lung CT volume and pulmonary function tests in a large interstitial lung diseases population. Then, we evaluate the longitudinal CT volume loss between IPF and non-IPF populations and explore a predictive value of annual CT volume loss in the IPF population.

## Materials and methods

### Study population

We conducted a retrospective, observational, longitudinal study between February 2005 and July 2018 in an ILD expert center (Louis Pradel Hospital, Hospices Civils de Lyon, Lyon, France). Clinical, functional, and imaging data for consecutive patients that underwent at least one unenhanced HRCT study for fibrotic ILD were collected. The diagnosis of ILD was made in multidisciplinary discussion according to international guidelines at the time of the patient’s presentation. Data usage policy of the “Hospices Civils de Lyon” in terms of confidentiality, anonymization, and security was applied for each study, and approval was obtained from our local committee. Institutional review board approval was obtained for the study, and patient consent was waived.

### CT studies

For the comparison between the PFT and CT volume (CT*vol*), only patients having a CT study within the two weeks of the PFT were considered eligible. For the longitudinal CT*vol* loss evaluation within non-IPF and IPF groups, patients who underwent more than four CT examinations were included. Finally, for the predictive analysis of CT volume loss in IPF patients, patients who had a baseline CT and a 1-year follow-up CT (± 10 days) were included. For all CT examinations, patients with a history of recent (within 3 months) acute exacerbation, pneumothorax, pleural effusion, or lower respiratory tract infection and confirmed by two senior radiologists (with 6 and 20 years of experience in chest imaging, S.S-M. and L.B., respectively) were excluded in order to be representative of the chronic disease course of these fibrotic ILDs.

### CT protocols

All HRCT acquisitions were performed at the end of deep inspiration. The data were collected retrospectively from all CT examinations on several systems over the years: GE Medical Systems (Revolution GSI), Philips (Brilliance 40, Brilliance 64, iCT 256, Ingenuity CT, IQon, Spectral CT), Siemens (Somatom Definition AS and AS +). The scanning parameters were as follows: tube voltage = 100–140 kVp (mean ± SD: 121 ± 9 kVp), helical scan mode. The mean slice thickness was 1.8 ± 0.8 mm (range: 0.9–3 mm).

### Automatic quantification of lung CT*vol*

**Fig. 1:**
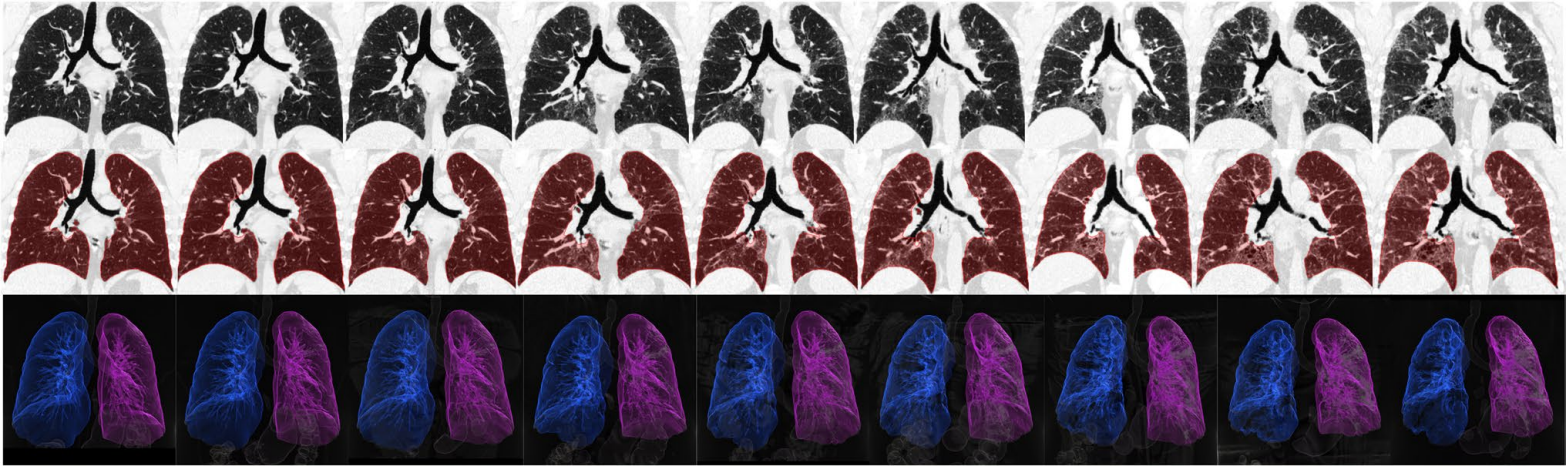
Automated segmentation of 9 CT lung volumes over a 6-year follow-up in a 58-year-old male affected by idiopathic pulmonary fibrosis. The first row represents the coronal images. The second row represents the mask segmentation of the lung. The third row represents the 3D volume after lung segmentation using commercially available software based on a U-net-based deep learning algorithm (CT Pulmo Auto Results un-released, provided under a research contract; IntelliSpace Portal ISP11.1, Philips Healthcare)

We used commercially available software implemented in a clinical workstation (CT Pulmo Auto Results, provided under a research contract; IntelliSpace Portal ISP11.1, Philips Healthcare). This software is a U-net-based deep learning algorithm and allows the lung segmentation with the exclusion of the main airways including the trachea, stem, lobar bronchi, and the main vessels. Lung CT*vol* was expressed in liters (L) (Fig. 1).

### Clinical respiratory functional test data

Patients’ data on demographics and PFTs were collected from patients’ records. PFTs were performed in all patients according to ATS/ERS official statement [16]. Total lung capacity (TLC) was measured with body plethysmography, and forced vital capacity (FVC) by spirometry, both expressed in liters (L).

### Statistical analysis

The data are expressed as mean ± standard deviation (SD) and median with interquartile range (IQR) for continuous variables. Categorical variables were presented as frequency (percentage). Distributions were tested for normality using the D’Agostino-Pearson test. Variables were compared using two paired Student t-test or Wilcoxon rank-sum test, as appropriate. For comparison purposes, differences in lung CT*vol* and functional volumes were compared using the Wilcoxon matched-pairs signed-rank test. For comparison between CT*vol* and respiratory volumes (FVC and TLC), a Bland–Altman analysis (bias, limits of agreement), a linear regression analysis (95% confidence interval, *R*^2^), and Spearman correlation coefficients and their 95% confidence were calculated.

For estimation of the daily and annual CT*vol* loss during follow-up, linear regression between all individual CT across time was calculated. The Mann–Whitney test was used to compare daily and annual CT*vol* loss between IPF and non-IPF groups.

For determining a predictive CT*vol* loss in the IPF population for major adverse events (MAE), i.e., death and transplantation, we first calculated the absolute annual and relative CT*vol* loss between CT baseline and 1-year follow-up CT of each patient that underwent a minimal 3-year follow-up or had died or undergone transplantation within 3 years. Then, a receiver operator characteristic (ROC) analysis was used to evaluate the greater baseline CT*vol* and CT*vol* loss values with the Youden index after having dichotomized the population with or without MAE at 4 years after the first CT study (baseline). Accordingly, to this value, a chi-square test with Yates’ continuity correction was used to test the association between categorical variables (MAE at 4 years, baseline CT*vol*, annual and relative CT*vol* loss). Univariate Cox regressions were performed. A Kaplan–Meier statistical analysis was used to test the survival rate in the IPF population with the best predictor greater and lower than the threshold tested.

Statistical analysis was performed using the SPSS® software v23 (IBM) and R software v3.5. A *p* value less than 0.05 was considered significant. The Bonferroni correction was used to adjust the *p* values in the longitudinal and predictive studies, i.e., less than 0.01, respectively.

## Results

### Study population

**Fig. 2:**
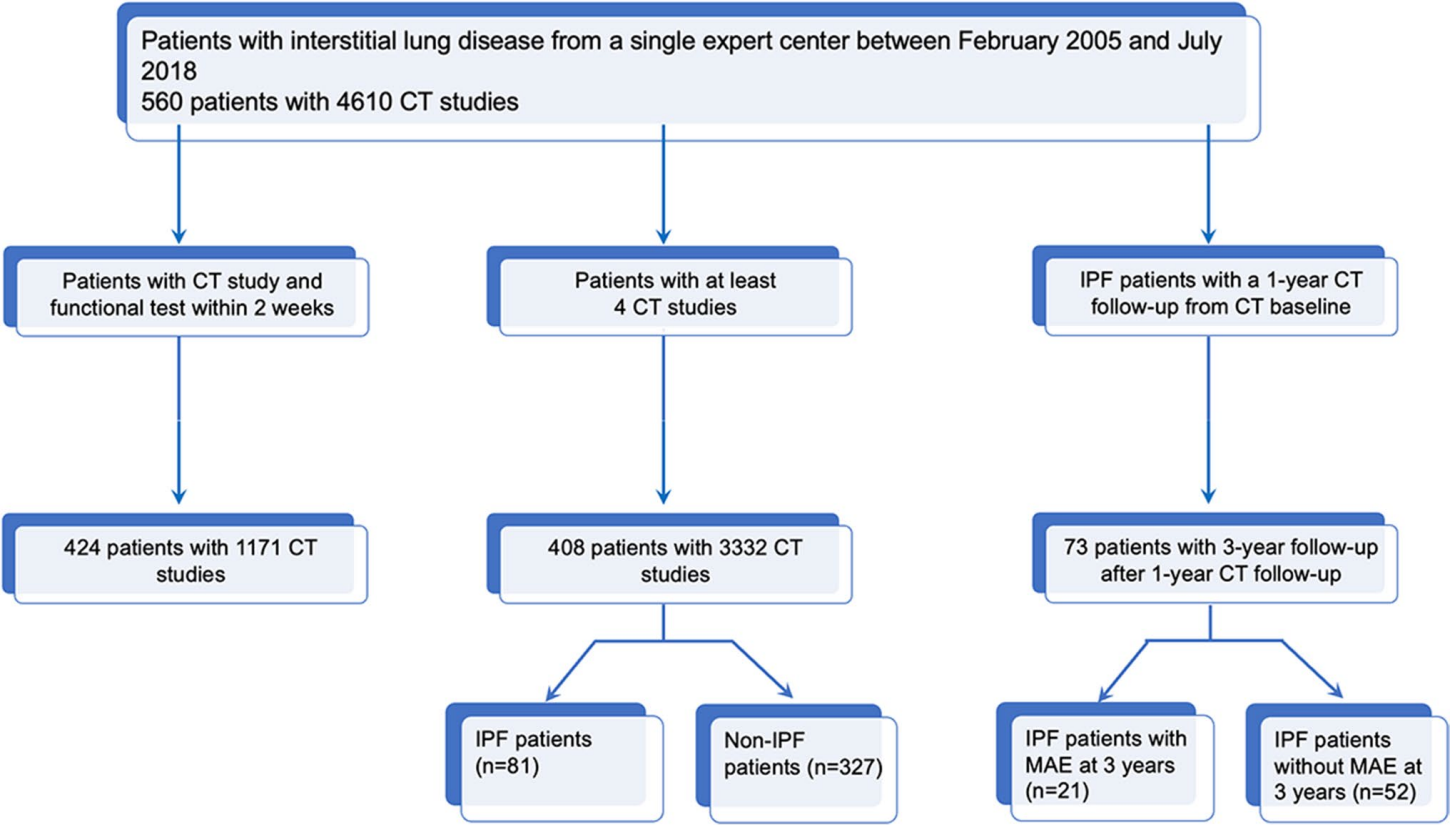
Study flow chart. Three different yet partly overlapping groups of patients were consecutively selected from the population of 560 patients: 296 patients are common to the first two groups; 53 patients are common to the last two groups

In total, 560 patients were included in the study (341 men (61%); mean age of 65.4 ± 13.9 years), corresponding to 4657 CT studies (mean CT studies per patient of 6.2 ± 4.6) (Fig. 2).

### Relation between CT*vol* and pulmonary function tests

**Fig. 3:**
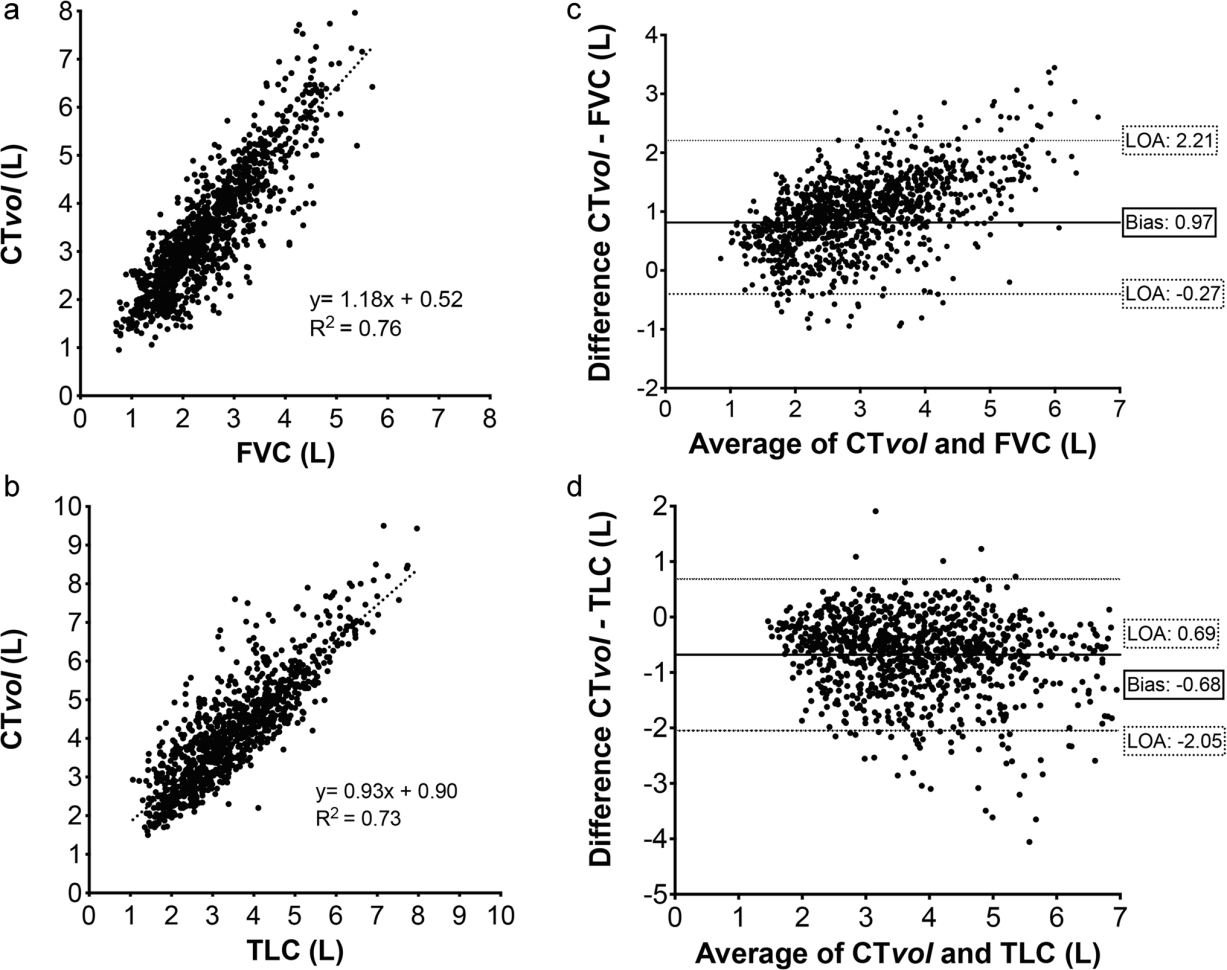
Linear regression and Bland–Altman analysis graphs between CT volume (CT*vol*), forced vital capacity (**a**, **b**) and total lung capacity (**c**, **d**)

We analyzed 424 patients corresponding to 1171 CT studies (Table 1). There was a good correlation between CT*vol* and FVC (Spearman coefficient of 0.86 (IC95%: 0.84–0.87, *p* < 0.0001) as well as between CT*vol* and TLC (Spearman coefficient of 0.84 (IC95%: 0.82–0.86, *p* < 0.0001) (Table 2). Bland–Altman analysis between CT*vol* and FVC revealed a positive proportional bias of 0.97, with 95.0% limits of agreement from − 0.27 to 2.21 L and a negative proportional bias between CT*vol* and TLC of − 0.68, with 95.0% limits of agreement from − 2.05 to 0.69 L (Table 2 and Fig. 3).

**Table 1:** Population characteristics of the comparative study

**Population number (*n*)**	424
Total CT studies	1171
CT studies per patient, mean ± SD	2.8 ± 1.8
Age (year-old), mean ± SD	64.6 ± 13.3
Sex (male), *n* (%)	256 (60.4)
**Interstitial lung disease type, *n* (%)**	
Idiopathic pulmonary fibrosis	108 (25.5)
Unclassifiable ILD	74 (17.4)
Systemic sclerosis	64 (15.1)
Combined pulmonary fibrosis and emphysema	46 (10.8)
Interstitial pneumonitis with auto-immune features	37 (8.7)
Dermatomyositis ILD	25 (5.9)
Hypersensitivity pneumonitis	22 (5.2)
Sjögren syndrome	19 (4.5)
Rheumatoid arthritis	14 (3.3)
Other (fibrotic COP, pneumoconiosis)	11 (2.6)
Sarcoidosis	4 (0.9)

Footnote. *n* is the patient number, *COP* cryptogenic organizing pneumonia, *ILD* interstitial lung disease

**Table 2:** Comparison between lung CT volume and pulmonary function tests (forced vital capacity and total lung capacity) in patients with fibrotic interstitial lug diseases (results of 1171 pairs of CT analyzed)

Analysis	Parameters	CT*vol* (L)	Forced vital capacity (L)	Total lung capacity (L)
	Median (IQR)	3.3 (2.6–4.3)	2.4 (1.8–3.1)	4.0 (3.1–5.0)
Linear regression statistics	Slope		1.18	0.93
	Offset		0.52	0.90
	R^2^		0.76	0.73
Correlation statistics	r		0.86	0.84
	95% CI		0.84–0.87	0.82–0.86
Bland–Altman statistics	r (95% CI)		0.86 (0.84–0.87)	0.84 (0.82–0.86)
	Bias (SD)		0.97 (0.63)	-0.68 (0.70)
	95% limits of agreement		− 0.27; − 2.21	− 2.05; − 0.69
	Slope (95% CI)		0.84 (0.79–0.95)	− 0.3 (− 0.40 to − 0.19)

Footnote. *FVC* forced vital capacity, *TLC* total lung capacity, *SD* standard deviation, *CTvol* CT volume, *LOA* limits of agreement

### Longitudinal lung CT*vol* loss between IPF and non-IPF groups

**Fig. 4:**
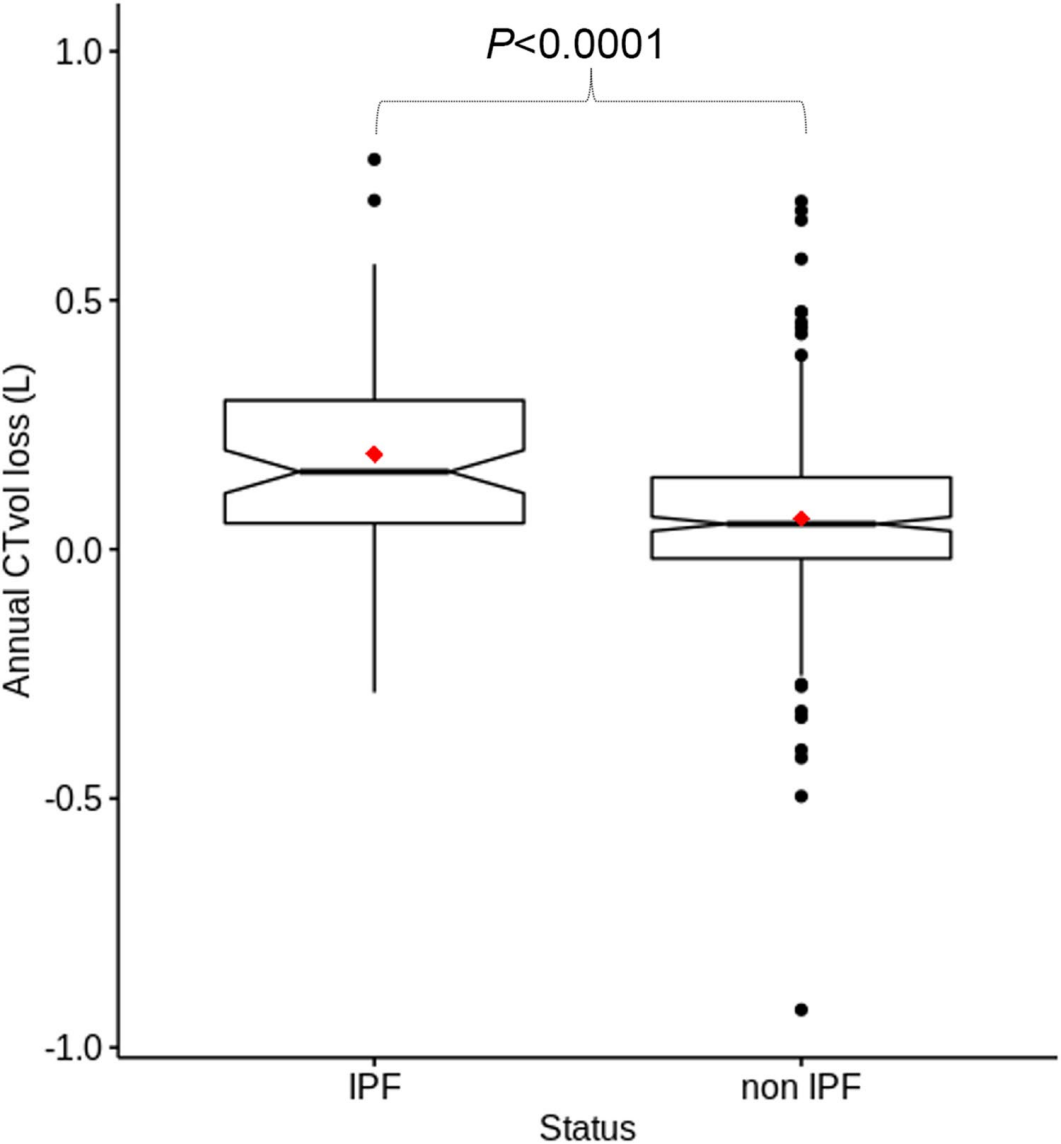
Notched box-and-whiskers plot showing the annual CT Volume (CT*vol*) in IPF and non-IPF groups. The lower and upper margins of each box indicate the 25th and 75th percentile. Median is marked by the line in the box, and mean by the red dot. 95% confidence interval of the median is represented by the notches and outliers indicate the minimal and maximal values

**Fig. 5:**
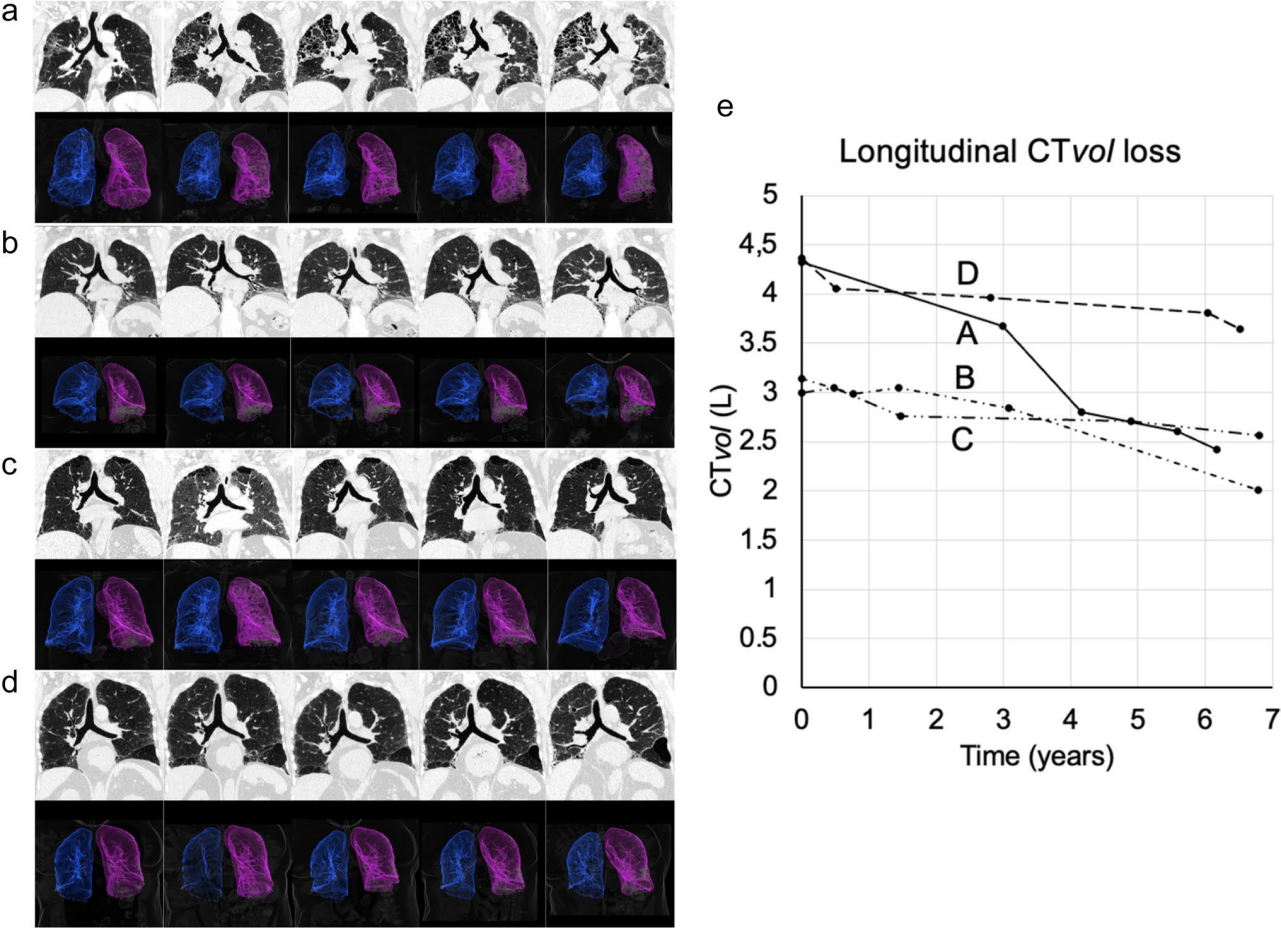
Representative cases of interstitial lung diseases over 6-year follow-up. (**a**) Idiopathic pulmonary fibrosis, (**b**) systemic sclerosis, (**c**) unclassifiable interstitial lung disease, (**d**) combined pulmonary fibrosis and emphysema, (**e**) individual longitudinal CT volume loss graph

We analyzed 408 patients corresponding to 3332 CT studies, which were distributed in two groups: 81 (19.9%) patients in the IPF group and 327 (80.1%) in the non-IPF group (Table 3, Figs. 4 and 5). Median (IQR) follow-up was 1345 (1034–1867) days (approximately 3.7 years) in the IPF group and 2051 (1456–2881) days (approximately 5.7 years) in the non-IPF group. Patients with IPF had a mean ± SD of 8.0 ± 3.6 CT studies while patients with non-IPF had a mean ± SD of 8.2 ± 3.7 CT studies. Median (IQR) daily CT*vol* loss was 0.41 mL (0.05–0.80) in IPF group and 0.14 mL (− 0.05–0.40) in the non-IPF group (*p* < 0.0001). Median (IQR) annual CT*vol* loss was 155.7 mL (49.9–304.5) versus 50.7 mL (− 19.0–144.7), respectively (*p* < 0.0001) (Table 2).

**Table 3:** Population characteristics of the longitudinal study

	IPF group (*n* = 81)	Non-IPF group (*n* = 327)	ALL (*n* = 408)	*p*
CT studies	627	2705	3332	
CT studies per patient, mean ± SD	8.0 ± 3.6	8.2 ± 3.7	8.2 ± 3.6	
Age (year-old), mean ± SD	68.3 ± 0.6	60.89 ± 14.17	62.4 ± 13.7	< 0.0001
Sex (male), *n* (%)	67 (82.7)	167 (50.7)	233 (57.1)	
Follow-up (days), median (IQR)	1345 (1034–1867)	2051 (1456–2881)	1819 (1301–2697)	< 0.0001
Interstitial lung disease type, ***n*** (%)				
Idiopathic pulmonary fibrosis			81 (19.9)	
Unclassifiable ILD			69 (16.9)	
Systemic sclerosis			72 (17.6)	
Combined pulmonary fibrosis and emphysema			44 (10.8)	
Interstitial pneumonitis with auto-immune features			35 (8.6)	
Dermatomyositis ILD			31 (7.6)	
Hypersensitivity pneumonitis			21 (5.1)	
Rheumatoid arthritis			19 (4.6)	
Sjögren syndrome			15 (3.7)	
Other (fibrotic COP, pneumoconiosis)			15 (3.7)	
Sarcoidosis			6 (1.5)	
Daily CT*vol* loss (mL), median (Q1–Q3))	0.41 (0.05–0.80)	0.14 (–0.05–0.40)	0.16 (–0.01–0.46)	< 0.0001
Annual CT*vol* loss (mL), median (Q1–Q3)	155.7 (49.9–304.5)	50.7 (–19.0–144.7)	58.9 (–5.5–167.8)	< 0.0001

Footnote. n is the patient number, COP: cryptogenic organizing pneumonia, ILD: interstitial lung disease. Values are expressed as median and (IQR) or mean ± SD, as appropriate

### One-year lung volume loss from baseline in the IPF group

Annual CT*vol* loss between the CT baseline and the 1-year CT follow-up of 73 patients with IPF were analyzed. Twenty-one MAEs during the 3-year follow-up period were reported with a mean ± SD delay of 440 ± 288 days after the annual CT. Sixteen patients died with a mean ± SD delay of 416 ± 288 days after the annual CT from pneumonia (6, 38%), respiratory failure (3, 19%), acute exacerbation (4, 25%), lung cancer (1, 6%), sepsis (1, 6%), and pulmonary embolism (1, 6%). Five patients underwent single lung transplantation with a mean ± SD delay of 542 ± 197 days after the annual CT. Eight patients were lost before the 3-year follow-up and were previously excluded from the initial population. Four patients underwent transplantation before the annual CT follow-up and were excluded from the predictive analysis. Dichotomized baseline characteristics of the study population by presence of MAE are summarized in Table 4.

**Table 4:** Characteristics of patients with idiopathic pulmonary fibrosis

	No major adverse event at 3-year follow-up (*N* = 52)	Major adverse event at 3-year follow-up (*N* = 21)	All (*n* = 73)	*p*
Age at baseline (years)				> 0.05
Mean (SD)	71 (7)	66 (13)	69 (9)	
Median (Q1–Q3)	70 (68, 76)	70 (58, 73)	70 (65, 76)	
Min–Max	50–82	36–88	36–88	
Sex				> 0.05
Male	43 (82.7%)	18 (85.7%)	61 (83.6%)	
Follow-up period (days)				< 0.001
Mean (SD)	1095 (0)	440 (336)	907 (347)	
Median (Q1–Q3)	1095 (1095, 1095)	517 (115, 721)	1095 (841, 1095)	
Min–max	1095–1095	12–1006	12–1095	
Baseline CT*vol* (L)				> 0.05
Mean (SD)	3.425 (1.051)	3.147 (0.898)	3.345 (1.011)	
Median (Q1, Q3)	3.316 (2.658, 3.906)	3.131 (2.614, 3.678)	3.304 (2.614, 3.896)	
Min–max	1.835–6.668	1.480–4.977	1.480–6.668	
Absolute annual CT***vol*** loss (L)				0.003
Mean (SD)	0.113 (0.620)	0.559 (0.538)	0.242 (0.627)	
Median (Q1–Q3)	0.048 (− 0.242, 0.455)	0.488 (0.367, 0.772)	0.275 (− 0.104, 0.575)	
Min–max	− 1.171 to 2.096	− 0.670 to 1.657	− 1.171 to 2.096	
Relative annual CT***vol*** loss (%)				0.001
Mean (SD)	2.07 (19.06)	17.44 (18.87)	6.49 (20.13)	
Median (Q1–Q3)	1.55 (− 7.21, 13.93)	16.87 (9.29, 28.05)	6.32 (− 2.61, 19.96)	
Min–max	− 52.14 to 50.10	− 33.40 to 51.16	− 52.14 to 51.16	

Footnote: *CTvol *CT volume loss calculated between the baseline CT and the 1-year follow-up CT, *SD* standard deviation. Values are expressed as median and (IQR) or mean ± SD, as appropriate. Chi-square and Kruskal–Wallis tests performed on proportions or continuous variables respectively. *p* values less than 0.01 were considered significant using the Bonferroni correction

**Fig. 6:**
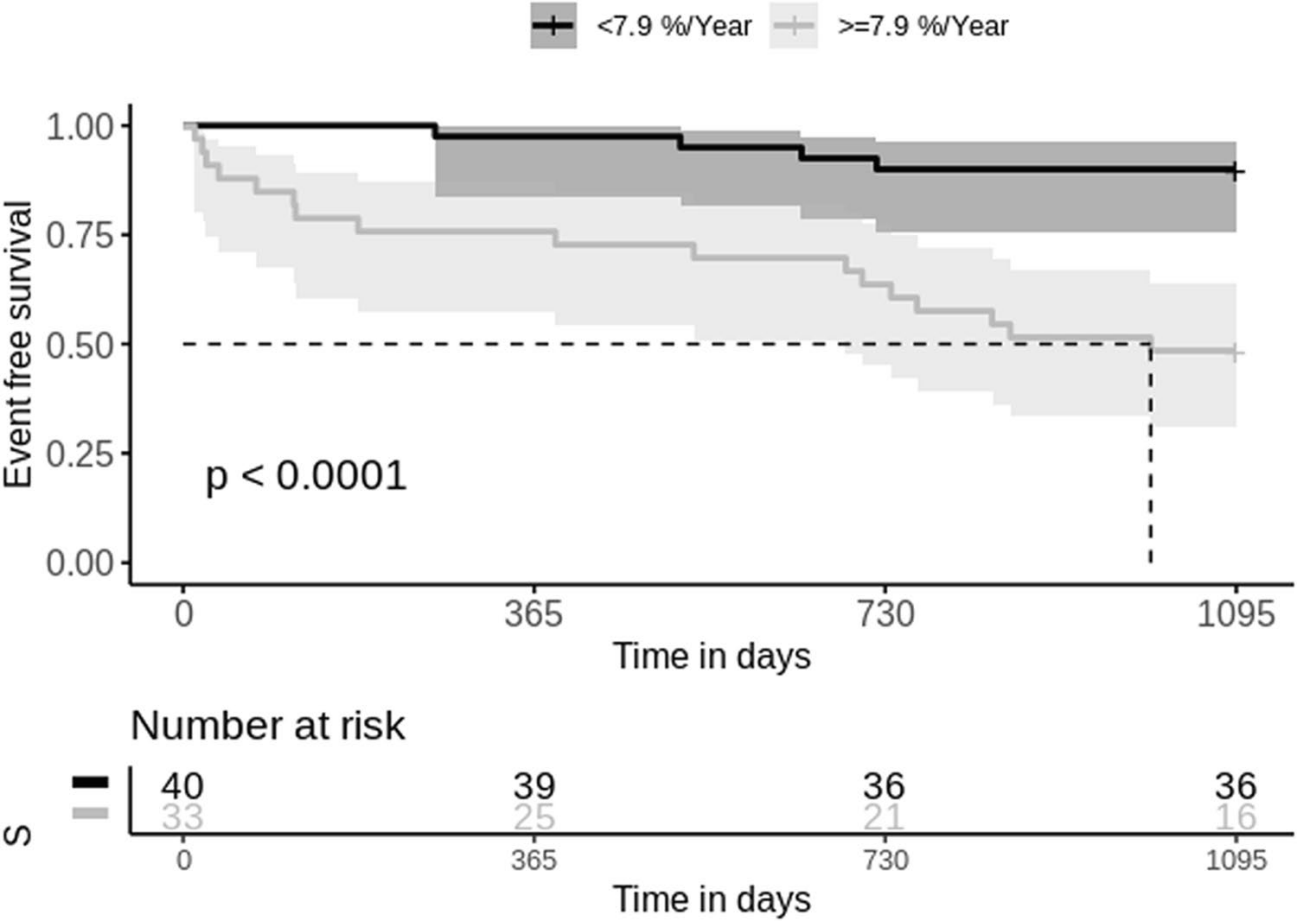
Three-year survival Kaplan–Meier curve and log-rank test, as a function of a 7.9% threshold for the relative CT*vol* loss in the idiopathic pulmonary fibrosis population. Mean survival times are 1039 (SEM: 28) and 746 (SEM: 73) days respectively (2.8 versus 2.0 years)

Based on a ROC statistical analysis, performed on baseline CT*vol*, absolute and relative annual CT*vol* loss, we found that a relative CT*vol* loss of 7.9% best matched for greater sensitivity and specificity, respectively of 81.0% and 69.2%, with an AUC at 0.74 [0.60; 0.86]. Optimal threshold for absolute CT*vol* loss was 0.37 L/year (76.2% sensitivity and 71.1% specificity, AUC = 0.73 [0.60–0.84]) and baseline CT*vol* was 3.19 L (57.1% sensitivity and 61.5% specificity, AUC = 0.56 [0.41–0.71]). Using multiple univariate Cox regression models (Table 5), no difference was observed with respect to age, sex, and baseline CT*vol*. But we found a significant effect for annual absolute CT*vol* loss (*p* < 0.01) and relative annual CT*vol* loss (*p* < 0.001), both dichotomized, on patient survival. The categorical variable (death at 4 years) was significantly associated with the annual CT*vol* loss with a *p* < 0.001 using a chi-square test with Yates’ continuity correction. Log-rank test demonstrated a significant association as well with a *p* < 0.0001. Kaplan–Meier survival curves are plotted in Fig. 6 according to 7.9% threshold for relative annual CT*vol* loss. Mean survival times were 1039 (SEM: 28) and 746 (SEM: 73) days respectively in the groups with lower and higher relative CT*vol* loss than 7.9%.

**Table 5:** Univariate Cox regression models: CT*vol* parameters are dichotomized (*) after application of separate thresholds (baseline of 3.19 L, decline of 0.37 L or 7.9% per year from baseline). Cox regression was significant for absolute and relative CT*vol* loss

	beta	HR (95% CI	Wald.test	*p*
Age (years)	− 0.049	0.95 (0.91–0.99)	5.1	0.02
Sex (female vs male)	0.14	1.2 (0.34–3.9)	0.05	> 0.05
Baseline CTvol*	0.52	1.7 (0.72–4.0)	1.4	> 0.05
Annual CT*vol* loss*	1.5	4.7 (1.8–12)	10	0.0014
Relative annual CT*vol* loss*	1.9	6.8 (2.3–20)	12	0.0006

*p* values less than 0.01 were considered significant using the Bonferroni correction

## Discussion

In the present study, we found that lung CT volume measurement, enabled by an automatic approach based on a deep learning algorithm, correlated strongly with FVC and TLC. Using longitudinal lung CT volume loss, we found that patients with IPF had a distinct disease course than other ILDs. In addition, in the IPF group, higher annual CT volume loss was associated with a worse prognosis.

The first results were the colinear relationship between CT*vol* with TLC, as previously shown [14, 17], but also with FVC among 1171 pairs of CT and PFT in a large cohort of ILD patients. CT*vol* was lower than TLC, such as previously demonstrated [18], which can be explained by patient positioning, e.g., the difference between CT exams (decubitus) and plethysmography (sitting position). Moreover, TLC measurement (body plethysmography) sums up all thoracic airspaces (including anatomic dead space) while CT*vol* only provides the measurement of parenchymal lung volume [19]. Thus, the delta between TLC and CT*vol* represents an anatomic dead space of 450 mL. CT*vol* on the other hand, was higher than FVC, which can be explained by the contribution of the residual volume contrary to the evaluation of FVC. In addition, the Bland Altman analysis showed a positive proportional bias suggesting a higher difference between CT*vol* and FVC for high lung volumes.

From a clinical perspective, longitudinal assessment of volume loss may be more useful than a single measurement at baseline. The results of the present longitudinal study demonstrated a greater and faster CT*vol* loss among IPF than non-IPF patients, associated with shorter follow-up time (due to greater mortality) but a comparable number of CT exams. These findings are consistent with higher morbidity and mortality in IPF as compared to other fibrotic ILDs, leading to more frequent acute complications requiring more exams, and earlier death [20]. In addition, results from the longitudinal analysis showed in the IPF population a good agreement between annual CT*vol* loss and annual FVC loss as reported previously [21]. Of note, PFTs are time-consuming and require multiple measurements along with technical expertise in order to be reproducible [22–27].

Finally, in our attempt to clarify the interest for lung CT*vol* in the IPF population, we showed a significant association between the annual CT*vol* loss and the prognosis. We found a relative CT*vol* loss predictive value of 7.9% for death at 3 years after 1-year CT follow-up which is close to the 10% FVC decline threshold currently used as an endpoint, i.e., indicating disease progression in IPF clinical studies [28]. Using the 7.9% threshold, we found a mean survival time of 2.8 versus 2.0 years which is in line with the average 2.5-year survival time reported in IPF patients [29]. Interestingly, we found also an association between the absolute CT*vol* loss and mortality. In addition, the baseline CT volume was not associated with the prognosis contrary to the CT volume loss while there was a trend to a lower baseline CT in patients with poor prognosis. This result is supporting the findings of previous studies that highlighted the stronger prognostic value of the clinical and physiological parameters change compared to the baseline lung function in fibrotic ILD [30, 31]. However, the meaning of this finding may be limited due to the absence of normalization as a function of the age, sex, and height of the patient.

In this study, the strength was to use an automated solution based on a deep learning algorithm that allowed the analysis of a large cohort, previously limited by exclusive manual processing [21–23]. This solution is a commercially available fully automatic application that does not require any user interaction, besides installation, and that is part of the software suite available in the IntelliSpace Portal (Philips Healthcare). In our study, this was made available as a standalone to be deployed in a batch mode. But lung volumetric measurements can be done on-demand or in pre-processing mode as soon as the data from the scanner is available. Running time (including additional measurements and reporting) varies, depending on the IT network and system workload, typically less than 2–3 min. Analysis of the inter-observer variability between a manual and automatic segmentation reported by the vendor showed a median and interquartile range (IQR) of the absolute volume differences of 19.6 ml and 9.1–32.0 mL IQR (Q1–Q3) [32], which appears as a relatively small range in comparison to the CT*vol* loss values reported in our study. Taken together, this explains the feasibility for investigating lung volumes at CT in a large representative cohort of consecutive patients with fibrotic ILD in a center of expertise. In addition, this automatic approach may be of great interest in many cases where measuring PFTs can be challenging. PFTs, although generally reproducible, are also person-dependent (i.e., on the patient and on the technician or physician performing the test). Measuring lung volumes using a different method could help interpret the findings, as do complementary methods in other areas. One additional result from the CT that cannot be obtained by PFTs is the ability to measure lobar volumes that may help to define the clinical evolution in some fibrotic ILDs, as suggested recently in pleuroparenchymal fibroelastosis [33]. Hence, our study represents a first step towards the identification of a new biomarker predicting physiological outcomes in order to consider the design of a new IPF mortality-risk score [34]. By taking into account additional CT parameters, automatic quantitative CT analysis could become a valid alternative or a complementary tool to pulmonary function tests in patients with ILD. However, the implementation of this technique in clinical practice warrants further prospective and controlled studies.

This study besides its retrospective and monocentric design has several limitations. First, CT*vol* measurement may vary with the individual’s degree of inspiration during the CT examination. However, previous study reported an acceptable variability (< 10%) and a good repeatability of CT*vol* in patients with restrictive lung diseases, explained by reduced pulmonary compliance leading to less variable inspiratory volumes [36]. Hence, we assumed that inspiratory CT would be sufficient for patient follow-up although combined inspiratory and expiratory CT exams are often recommended [37]. Second, the presence of concomitant emphysema in ILD can underestimate disease progression despite FVC and TLC remaining stable [7], that is why estimation of lung volume solely with PFT can therefore sometimes be insufficient to monitor restrictive physiology. Nevertheless, our study did not take into account patients with concomitant emphysema. Hence, it would be interesting to perform additional lobar segmentation since pulmonary fibrosis takes place mainly at the basal lobes while emphysematous changes preferentially affect upper lobes [38]. Third, in the longitudinal study, patients with at least 4 CT studies were included to guarantee the quality of linear regressions which may be variable depending on the sampling pattern across time, i.e., may lead to influential points. However, in this real-life study, the CTs scans were not performed at fixed intervals, which can represent a bias. Fourth, our comparative study between the PFT results and CT volume values did not take into account the PFT values expressed as a percentage of the predicted value. Nevertheless, our aim was to evaluate the absolute precision of the CT for volume quantification. Fifth, the annual CT volume loss was assessed only in the IPF patients. Currently, there is no recommendation for performing an annual CT follow-up in the non-IPF patients, contrary to the IPF patients who undergo annual follow-up CT in our expert center according to the French recommendations for the management of IPF [39]. This explains the difficulty to assess the annual CT lung volume loss in non-IPF patients. In addition, non-IPF patients who had a follow-up CT probably had a CT due to a clinical indication including the occurrence of acute exacerbations, which may bias our analysis of the chronic disease course of the fibrotic ILD. Finally, cases were enrolled over a period of 13 years, and several different CTs were used; this in fact demonstrates that our approach can be implemented to various scenarios in a real-life setting.

In conclusion, automatic evaluation of the lung CT volume, in patients with ILD and particularly with IPF, may be an alternative or a complementary biomarker to pulmonary function tests for assessment of lung volume loss, in clinical care as in randomized trials.

## Abbreviations

CT: Computed tomography
CT*vol*: CT volume
FVC: Forced vital capacity
HRCT: High-resolution computed tomography
ILD: Interstitial lung disease
IPF: Idiopathic pulmonary fibrosis
MAE: Major adverse event
PFT: Pulmonary function test
TLC: Total lung capacity

## Declarations

### Guarantor

The scientific guarantor of this publication is Pr. Loic Boussel.

### Statistics and biometry

No complex statistical methods were necessary for this paper.

### Methodology

• Observational study
